# Pharmacy Students’ Knowledge and Attitude toward Registration Trials and Clinical Research: A Survey in a Japanese University Hospital

**DOI:** 10.3390/pharmacy5040067

**Published:** 2017-12-11

**Authors:** Natsuko Ise, Kenshi Takechi, Toshiko Miyamoto, Keisuke Ishizawa, Hiroaki Yanagawa

**Affiliations:** 1 Clinical Trial Center for Developmental Therapeutics, Tokushima University Hospital, Tokushima 770-8503, Japan; inatsuko@tokushima-u.ac.jp (N.I.); kenshi.takechi@tokushima-u.ac.jp (K.T.); t.miyamoto@tokushima-u.ac.jp (T.M.); 2 Division of Pharmacy, Tokushima University Hospital, Tokushima 770-8503, Japan; ishizawa@tokushima-u.ac.jp

**Keywords:** pharmacy students, clinical research coordinators, clinical research, attitude, Japan

## Abstract

Clinical research plays a fundamental role in establishing new treatments. Clinical research coordinators are considered essential in clinical research, and medical professionals such as pharmacists often take on this role. Pharmacy students can be considered future candidates for this task. We used questionnaires to survey the knowledge of and attitudes toward registration trials and clinical research of pharmacy students at Tokushima University Hospital. All pharmacy students (103) to whom questionnaires were sent responded. Almost all respondents were aware of registration trials and clinical research. More than 90% were aware of the existence of clinical research coordinators, and about half (48.6%) understood their role. In clinical research terminology, most respondents were aware of informed consent and related issues, but fewer than 20% were aware of more practical things. In total, 29.1% and 40.8% of the respondents were willing to carry out and coordinate research. These findings suggest that pharmacy students have basic knowledge of clinical research and that many students are willing to carry out and coordinate clinical research. More practical exposure to clinical research may help to strengthen their future contribution. Further studies may help to determine how to provide education on registration trials and clinical research to pharmacy students.

## 1. Introduction

Clinical research plays an important role in promoting the quality of medical practice, including the approval of drugs and medical devices. Trials for this purpose are referred to as “registration trials” in this article. The Japanese infrastructure for registration trials has improved since the introduction of the Good Clinical Practice standard in 1997, and the contribution of clinical research coordinators (CRCs) in registration trials is now widely recognized as providing practical assistance and quality assurance. In Japan, most registration trials conducted in clinics and hospitals are supported by CRCs employed by the site management organization, whereas those at academic hospitals, national centers and national hospital organizations are usually supported by a CRC employed by the institution [1]. In these major hospitals, pharmacists and nurses often act as CRCs. Pharmacy students and nursing students can therefore be considered as future CRCs.

The Japanese curriculum for pharmacy education was changed to a six-year program in 2006, to meet the requirements of an era of rapid advances in science and technology. Although drug development and registration trials were previously covered, there have been rapid changes in these areas. For example, the delay in approval of new drugs in Japan compared with the United States and other countries (the so-called “drug lag”) is a long-standing issue in Japanese clinical trials [2]. Recent reports showed that various strategies, such as inclusion of Japanese subjects in global clinical trials and better early trial design and planning, had successfully reduced the drug lag [3,4]. The need to improve education systems in Japanese universities, and introducing practical methods to educate students on registration trials and clinical research still remain to be addressed. Tokushima University’s new pharmacy practice program started in 2010, and two years later, the Clinical Trial Center for Developmental Therapeutics (CTCDT) at Tokushima University Hospital started to expose students on that course to experience of registration trials and the clinical research environment.

We have already reported physicians’ view on registration trials [5], and nurse awareness of clinical research [6]. Understanding pharmacy students’ knowledge of and attitude toward registration trials and clinical research may also contribute to the establishment of clinical research infrastructure, so this study focused on pharmacy students and used questionnaires to survey their attitudes and knowledge linked to experience of exposure to the research environment at Tokushima University Hospital.

## 2. Methods

### 2.1. Setting and Participants

Participants were students exposed to the registration trial environment by a pharmacist CRC as part of the pharmacy practice program of Tokushima University [7]. The pharmacy practice program itself is a patient-oriented education program started nationwide in Japan in 2010, and the details are planned at each university. In 2012, members of the CTCDT started to provide students with exposure to the registration trial environment, by involving pharmacist CRCs. All pharmacy students attend the exposure program by small groups in rotation. The exposure program includes a presentation about registration trials, a tour of the CTCDT (including the administrative office and monitoring room), and small-group discussions with a pharmacist CRC. From 2013 to 2015, a total of 103 pharmacy students went through this program, and completed the questionnaire for this study. This study was approved by the Ethics Committee of Tokushima University Hospital.

### 2.2. Questionnaire to Assess Pharmacy Students’ Knowledge of and Attitudes toward Registration Trials and Clinical Research

We assessed pharmacy students’ knowledge of and attitudes toward registration trials and clinical research in a cross-sectional study. Our questionnaire was based on one used in a previous study involving nurses [6] and revised to better fit pharmacy students. Pharmacy students are expected to be familiar with drug development process, so several questions about registration trials were added. Questions about nursing studies and nurse experience were removed. The revised questionnaire was tested on six CRCs from the CTCDT and further revised following their suggestions. The questionnaire was anonymous and contained six parts with 39 questions (see Supplementary File 1). The first part consisted of two demographic questions. The second part consisted of five questions to determine general awareness of registration trials, clinical research, and the role of the CRC. The third part consisted of nine questions about registration trials, and three about clinical research. The fourth part was new and consisted of two questions to survey pharmacy students’ general perceptions of registration trials. For the first question, students chose from “dark image,” “bright image,” “awful,” “difficult,” and “not familiar.” For the second question, students could select from “drug development,” “investigation,” “human experiments,” “treatment,” and “volunteers.” Pharmacy students were asked to select any terms that matched their general perception of registration trials. The fifth part consisted of 15 questions about research-related terminology. In addition to nine questions from the previous questionnaire, six new questions about practical terms related to registration trials were included in the revised questionnaire. The sixth part included three questions about respondents’ views on their need to learn more about registration trials and clinical research and willingness to act as investigators or CRCs, and to participate in a study if eligible. The first and third questions used a five-point Likert-type scale (strongly agree, agree, neutral, disagree, and strongly disagree), and the second question used a three-point scale of both (investigator and CRC), one, or neither. In questions to survey awareness in the second and fifth parts, a five-point Likert-type scale (confident, quite aware, aware, less aware, and not aware) was used. In the third part, participants were asked to tick a box if they were aware of the issue.

To assess changes following exposure to the research environment, another questionnaire (the post-exposure questionnaire) was developed (see Supplementary File 2). This was also anonymous and included the pre-exposure questionnaire’s first part (the two demographic questions), fourth part (the two questions to survey pharmacy students’ general perception of registration trials), and sixth part (three questions about their views on the need to learn more about registration trials and clinical research and their willingness to act as investigators, CRCs, and study participants). A question was added to survey views on a practical method of educating students. The question was “I think it would be effective for my career to learn about registration trials by a more practical method, such as role playing” and responses used a five-point Likert-type scale (strongly agree, agree, neutral, disagree, and strongly disagree).

The pre-exposure and post-exposure questionnaires were provided to pharmacy students before and after the exposure program by the pharmacist CRC involved. The CRC also explained that the survey was completely independent of their classes, participation was voluntary, and that refusal to participate would cause them no disadvantage.

### 2.3. Statystical Analysis

We compared general perceptions of registration trials and willingness to act as investigators or CRCs before and after the exposure program and analyzed the differences using the χ^2^ test. *p*-values < 0.05 were considered significant. All *p*-values were based on two-sided tests.

## 3. Results

### 3.1. Respondent Characteristics

All 103 pharmacy students completed questionnaires and were included in this analysis. The respondents included 41 males (39.8%) and 62 females (60.2%). The mean age ± SD of the respondents was 23.1 ± 1.73.

### 3.2. General Awareness of Registration Trials, Clinical Research, and the Role of the CRC

In Japan, registration trials are regulated by law. Other types of clinical research are based on the ethical guidelines of the Japanese ministries [1]. We therefore asked pharmacy students about their awareness of registration trials and clinical research and the differences between them.

Table 1 shows that almost all respondents were aware (confident, quite aware, or aware) of registration trials (100%) and clinical research (99.0%), but fewer were aware (confident, quite aware, or aware) of the differences between the two (64.1%). The vast majority (92.2%) of the respondents were aware (confident, quite aware, or aware) of the existence of CRCs, but fewer (48.6%) understood their role.

### 3.3. Awareness of Issues Related to Registration Trials and Clinical Research

Table 2 shows that most respondents were aware of some issues related to registration trials, particularly that “registration trials are necessary for drug registration,” “informed consent is essential for registration trials,” “refusal to participate in registration trials causes no disadvantage,” “some registration trials use placebo,” and “participants can withdraw anytime.” Fewer respondents were aware that “review by institutional review board is mandatory” (78.6%), “CRC support registration trials” (68.0%), “participants do not need to pay for drugs and tests related to registration trials” (73.8%), and “participants are given a reward for participating in registration trials” (55.3%). Around 60% of respondents were aware of the three issues related to clinical research.

### 3.4. General Perception of Registration Trials

**Figure 1:**
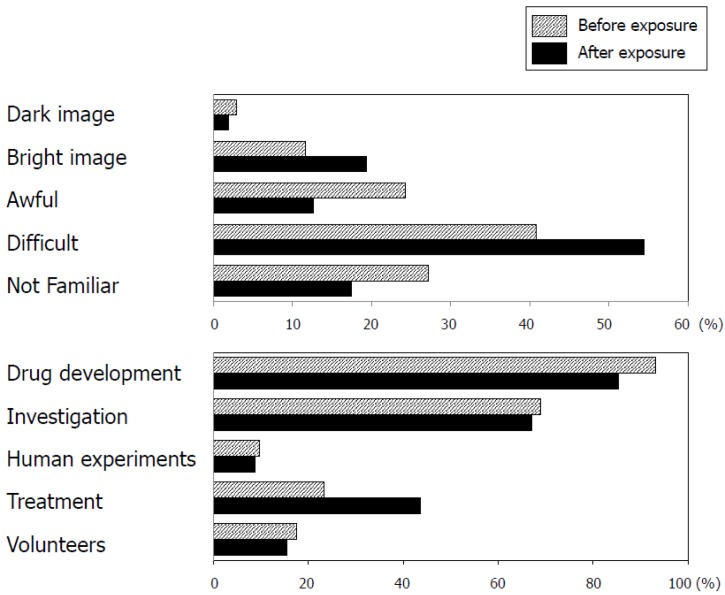
General perception of registration trials before and after exposure. Two questions were provided to pharmacy students before and after the exposure to the registration trial environment by a pharmacist CRC. Pharmacy students were asked to select any terms that matched their general perception of registration trials in each question.

Participants were asked to select terms that matched their general perception of registration trials in two questions, and the results are shown in Figure 1.

In the first question, although the percentage responding “difficult” increased after exposure, none of the differences were statistically significant.

In the second question, again, none of these differences were statistically significant.

### 3.5. Awareness of Research-Related Terminology

**Figure 2:**
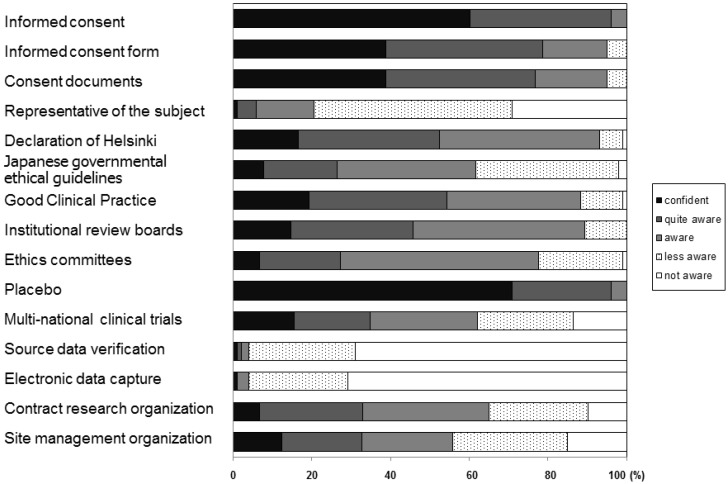
Awareness of research-related terminology.

We asked pharmacy students about their awareness of research-related terminology, and the results are shown in Figure 2. All respondents were aware (confident, quite aware, or aware) of the meaning of the term ‘placebo’. More than 90% of respondents were aware of informed consent and related issues (informed consent form and consent documents), Declaration of Helsinki, good clinical practice and institutional review boards. Fewer (50–80%) were aware of issues such as Japanese Governmental ethical guidelines (61.8%), ethics committees (77.7%), multi-national clinical trials (62.1%), contract research organizations (65.0%), and site management organizations (55.8%). Only 20.4% of respondents were aware of subject representatives, and very few were aware of source data verification (3.9%) and electronic data capture (3.9%).

### 3.6. Views on the Need to Learn More about Registration Trials and Clinical Research and Willingness to Act as Study Participants

Table 3 shows that almost all respondents (98%) agreed that they needed to learn more about registration trials and clinical research. Almost half (42.7%) said that they would be willing to participate in a registration trial if eligible. These percentages were slightly higher after the exposure program (99% and 48.6%), but these differences were not statistically significant. After the exposure program, we asked students about practical methods for educating them about clinical research and registration trials. Table 3 shows that the majority (67.7%) considered it effective to learn about registration trials by a more practical method, such as role playing.

### 3.7. Willingness to Act as Investigators and/or CRC

Table 4 shows that 29.1% of the respondents were willing to work as investigators and 40.8% as CRCs. These percentages remained stable after the exposure program (31.1% and 41.8%).

## 4. Discussion

Investigators running registration trials in Japan once performed all tasks related to the trial, from patient care to administrative work [8]. It is now widely accepted, however, that it is helpful to have a supporting division for clinical research, including a CRC. The role of the CRC was originally to reduce the workload of investigators, but now also contributes to the ethical conduct and quality assurance of registration trials. Their role in clinical research is still limited because of financial constraints. In 2013, a scandal involving several clinical trials of the anti-hypertensive drug valsartan made headlines in Japan and around the world [9,10]. This led to an ongoing review of Japanese systems of clinical research, including renewal of governmental ethical guidelines in 2015, and the establishment of a new law about clinical research. Investigator education is essential to prevent research misconduct. Increasing interest of other health professionals in registration trials and clinical research as well as establishing systems for health professionals to observe registration trials and clinical research could be useful strategies. Pharmacy students are future CRC candidates. In this study, we therefore focused on pharmacy students and surveyed their knowledge of and attitudes toward registration trials and clinical research.

Almost all respondents in this study were aware of registration trials and clinical research (Table 1), and of registration trial-related issues, such as those expressed as follows: “registration trials are necessary for drug registration,” “informed consent is essential for registration trials,” “refusal to participate in registration trials causes no disadvantage,” “some registration trials use placebo,” and “participants can withdraw anytime” (Table 2). Fewer respondents were aware of more practical issues in registration trials, such as those expressed as follows: “review by institutional review board is mandatory,” “CRC support registration trials,” “participants do not need to pay for drugs and tests related to registration trials,” and “participants are rewarded for participating in registration trials” (Table 2). Only 20.4% of respondents were aware of the existence of the term “subject representative,” and very few respondents were aware of source data verification (3.9%) and electronic data capture (3.9%) (Figure 2). In total, 64.1% of respondents were aware of the difference between registration trials and clinical research, and around 60% were aware of the three issues related to clinical research (Table 2). These results suggest pharmacy students have a good level of overall familiarity with the drug development and registration trial process, but less familiarity with the practical issues concerned with registration trials and clinical research. These possibilities should be examined in future studies.

As Table 1 shows, 92.2% of the respondents were aware (confident, quite aware, or aware) of the existence of CRCs, but fewer were aware of their role (48.6%), although 68.0% knew that CRCs support registration trials. Koyanagi et al. [11] reported on a survey of 314 pre- and post-graduate pharmacy students, and found that 43.5% had detailed knowledge of the role of CRCs, although 36.1% were only aware of the term. Our findings are in line with these previous results and suggest that the concept of CRCs is widely recognized, but their precise role is not widely understood among pharmacy students.

In total, 40.8% of our respondents were willing to act as a CRC in future, which was higher than a previous study on nurses [6]. Although education about the role of the CRC may affect willingness to take that role, but our exposure program had no effect on willingness (Table 3) or general perceptions (Figure 1).

Yoshida et al. reported that they used role playing in registration trial education in a pharmacy practice program [12]. They found that the most influential factor in enlarging interest in the CRC role was role playing of monitoring in registration trials.Arita et al. [13] and Imakyure et al. [14] also reported the usefulness of teaching the role clinical trials play. In our study, almost all respondents agreed that they needed to learn more about registration trials and clinical research, and the majority considered it would be effective to learn about more practical aspects of registration trials, such as the role they play (Table 3). Only a few respondents were aware of source data verification, so such practical education should be attempted to increase knowledge as well as improving perspectives of registration trials. We want to investigate these possibilities in future studies.

As well as being future CRCs, pharmacy students are also possible future clinical researchers. In the United States, the National Institute of Health has strongly endorsed the role of the clinical pharmacist as an investigator, and there has been a substantial increase in pharmacist-directed research, as shown by the growth in funded grants and scientific publications [15]. The American College of Clinical Pharmacy (ACCP) Research Institute conducted a Mentored Research Boot Camp, Focused Investigator Training (FIT) Program, and reported that this program was associated with a significant increase in attendees’ self-efficacy in obtaining external research funding [16]. Overholser et al. [17] reported that taking a two-credit elective course in clinical research increased pharmacy students’ interest in pursuing a career in clinical research. In this study, 29.1% of respondents were willing to work as investigators (Table 4). That seems to be an encouraging result, and we should build on this in Japan.

This study had several limitations. In particular, it was conducted in just one university hospital in Japan. Although almost all Japanese pharmacy students seem to have limited access to the registration trial and clinical research environment, this survey may not fully reflect the situation of pharmacy students in Japan. As the health system and clinical research infrastructure vary by country, the generalizability of our results in international settings should be examined in future studies.

## 5. Conclusions

Despite various limitations, we found that pharmacy students have considerable knowledge of registration trials and clinical research. Around 40% of respondents were willing to act as a CRC in the future. Due to these study limitations, further study is warranted to determine how pharmacy student education can contribute to establishing a suitable infrastructure for clinical research going forward.

## Figures and Tables

**Table 1:** General awareness of registration trials, clinical research, and the existence and the role of CRC.

	Confident	Quite Aware	Aware	Less Aware	Not Aware
Registration trials	25 (24.3%)	69 (67.0%)	9 (8.7%)	0 (0.0%)	0 (0.0%)
Clinical research	15 (14.6%)	70 (68.0%)	17 (16.5%)	1 (1.0%)	0 (0.0%)
Difference between registration trials and clinical research	3 (2.9%)	23 (22.3%)	40 (38.8%)	34 (33.0%)	3 (2.9%)
Presence of CRC	9 (8.7%)	49 (47.6%)	37 (35.9%)	84 (7.8%)	0 (0.0%)
Role of CRC	1 (1.0%)	11 (10.7%)	38 (36.9%)	51 (49.5%)	2 (1.9%)

**Table 2:** Awareness of issues related to registration trials and clinical research.

	Aware	Not Aware
1. Awareness of issues related to registration trials		
Registration trials are necessary for drug registration	101 (98.1%)	2 (1.9%)
Review by institutional review board is mandatory	81 (78.6%)	22 (21.4%)
CRC support registration trials	70 (68.0%)	33 (32.0%)
Informed consent is essential for a registration trial	102 (99.0%)	1 (1.0%)
Refusal of a registration trial causes no disadvantage	101 (98.1%)	2 (1.9%)
Some registration trials use placebo	103 (100.0%)	0 (0%)
Participants can withdraw anytime	100 (97.1%)	3 (2.9%)
Participants need not to pay for investigational drugs and tests related to registration trials	76 (73.8%)	27 (26.2%)
Reward for participants is prepared in registration trials	57 (55.3%)	46 (44.7%)
2. Awareness of issues related to clinical research		
Clinical research includes research using labeled drugs	63 (61.2%)	40 (38.8%)
Review by ethics committee is mandatory	63 (61.2%)	40 (38.8%)
Governmental ethical guidelines are applied to clinical research	69 (67.0%)	34 (33.0%)

**Table 3:** Views on the need to learn more about registration trials and clinical research and willingness to be study participants before and after exposure to registration trial environment.

**1. Before Exposure**					
	**Strongly Agree**	**Agree**	**Neutral**	**Disagree**	**Strongly Disagree**
It is necessary for pharmacy students to know more about registration trials and clinical research.	59 (57.3%)	41 (39.8%)	1 (1.0%)	1 (1.0%)	0 (0%)
If it was suggested I participate in some registration trial if eligible for the trial, I would participate.	13 (12.6%)	31 (30.1%)	26 (25.2%)	29 (28.2%)	4 (3.9%)
**2. After Exposure**					
	**Strongly Agree**	**Agree**	**Neutral**	**Disagree**	**Strongly Disagree**
It is necessary for pharmacy students to know more about registration trials and clinical research.	59 (57.3%)	43 (41.7%)	0 (0%)	1 (1.0%)	0 (0%)
If it was suggested I participate in some registration trial if eligible for the trial, I would participate.	15 (14.6%)	35 (34.0%)	24 (23.3%)	22 (21.4%)	7 (6.8%)
I think it is effective for my career to learn about registration trials by more practical method, such as role playing.	27 (26.5%)	42 (41.2%)	22 (21.6%)	10 (9.8%)	2 (1.9%)

**Table 4:** Willingness to act as investigators and/or CRCs before and after exposure to registration trial environment.

	Investigator	CRC	None
pre-exposure	30 (29.1%)	42 (40.8%)	43 (41.7%)
post-exposure	32 (31.1%)	43 (41.8%)	42 (40.8%)

## Supplementary Materials

The following are available online at http://www.mdpi.com/2226-4787/5/4/67/s1.
